# Subacute Stroke Following Aortic Root Dissection: A Clinical Case Report

**DOI:** 10.7759/cureus.108542

**Published:** 2026-05-09

**Authors:** Sandar Le Aung, Thin Ei San, Mansimran Singh Dulay, Sirr Ling Chin, Jasbir Dulay

**Affiliations:** 1 Department of Respiratory Medicine, Southmead Hospital, Bristol, GBR; 2 Department of Acute Medicine, Dorset County Hospital, Dorchester, GBR; 3 Department of Cardiology, Royal Brompton and Harefield Hospitals, King's College, London, GBR; 4 Surgical High Dependency Unit, Southampton General Hospital NHS Foundation Trust, Southampton, GBR; 5 Acute Medicine Unit, Southampton General Hospital NHS Foundation Trust, Southampton, GBR

**Keywords:** aortic root dissection, chest pain, stroke, sudden-onset, vacant episode

## Abstract

Aortic root dissection is a medical emergency, usually presenting with chest pain/epigastric pain, with a high mortality rate of at least 20-30%. Early diagnosis is critical as early prompt intervention can reverse the mortality rate, which is estimated to increase by 1% to 1.4% in the first 48 hours. This case report highlights the diagnostic challenges of type A aortic root dissection if presented with non-specific symptoms, a high-mortality medical emergency. A 58-year-old presented with acute chest pain and a vacant episode but was initially discharged following unremarkable investigation results (ECG, troponin, and chest X-rays). Ten days later, he returned with expressive dysphagia secondary to a subacute cerebral infarct, leading to the eventual diagnosis of aortic root dissection initially seen on echocardiogram and later confirmed by CT angiography. The patient underwent the surgical repair but ultimately passed away from *Escherichia (E.) coli* sepsis postoperatively. Ultimately, this report reinforces the necessity of considering D-dimer or bedside transthoracic echo as part of a comprehensive initial assessment for suspected aortic root dissection, while simultaneously emphasising that definitive diagnosis and management planning rely on the dedicated CT angiography.

## Introduction

Aortic root dissection is a life-threatening cardiovascular emergency resulting from an intimal tear in the ascending aorta, allowing blood to enter the medial layer and create a false lumen. It is classified as Stanford Type A aortic dissection, a category that includes all dissections involving the ascending aorta regardless of distal extension, and which mandates urgent surgical management [1,2]. Untreated Type A dissections are associated with a mortality rate approaching 1-2% per hour in the first 24-48 hours, with overall in-hospital mortality reported at 20-30% [3].

Classically, patients present with sudden-onset, severe “tearing” chest pain radiating to the back; however, presentations may be highly variable. Atypical features include epigastric pain, syncope, stroke-like symptoms, heart failure due to acute aortic regurgitation, or painless dissection, particularly in elderly patients [4,5]. These atypical presentations contribute significantly to delayed or missed diagnoses, which are reported in up to one-third of cases [6].

Early diagnosis is critical, as prompt surgical repair within 48 hours dramatically improves survival, with contemporary series reporting operative mortality rates as low as 1-5% [7]. CT aortic angiography is the diagnostic modality of choice due to its high sensitivity and specificity, although transthoracic or transoesophageal echocardiography may reveal indirect signs such as intimal flaps, aortic root dilatation, or pericardial effusion [8]. Elevated D-dimer levels may support early risk stratification but are insufficient as a standalone diagnostic test [9].

This case report has been presented at the Society of Acute Medicine (SAM) Conference in Brighton, UK, in 2024.

## Case presentation

A 58-year-old man with a background of severe obstructive sleep apnoea managed with nocturnal continuous positive airway pressure (CPAP) presented to the emergency department with sudden-onset, severe central chest pain. The pain was described as constant, non-pleuritic, and radiating to the upper abdomen, with no clear exertional trigger. On initial assessment, his blood pressure was 168/92 mmHg, heart rate 96 beats/min, respiratory rate 18 breaths/min, oxygen saturation 98% on room air, and he was afebrile. Cardiovascular and respiratory examinations were documented as unremarkable, with no murmur appreciated at that time.

While in the emergency department, the patient experienced a transient episode of altered consciousness lasting approximately 20 minutes, described by staff as a vacant stare with reduced responsiveness but no tonic-clonic activity, focal neurological deficit, or post-ictal confusion. He subsequently returned to baseline. Initial investigations, including serial high-sensitivity troponins, electrocardiogram, and chest radiograph, were unremarkable. He was discharged home with oral analgesia.

**Figure 1:**
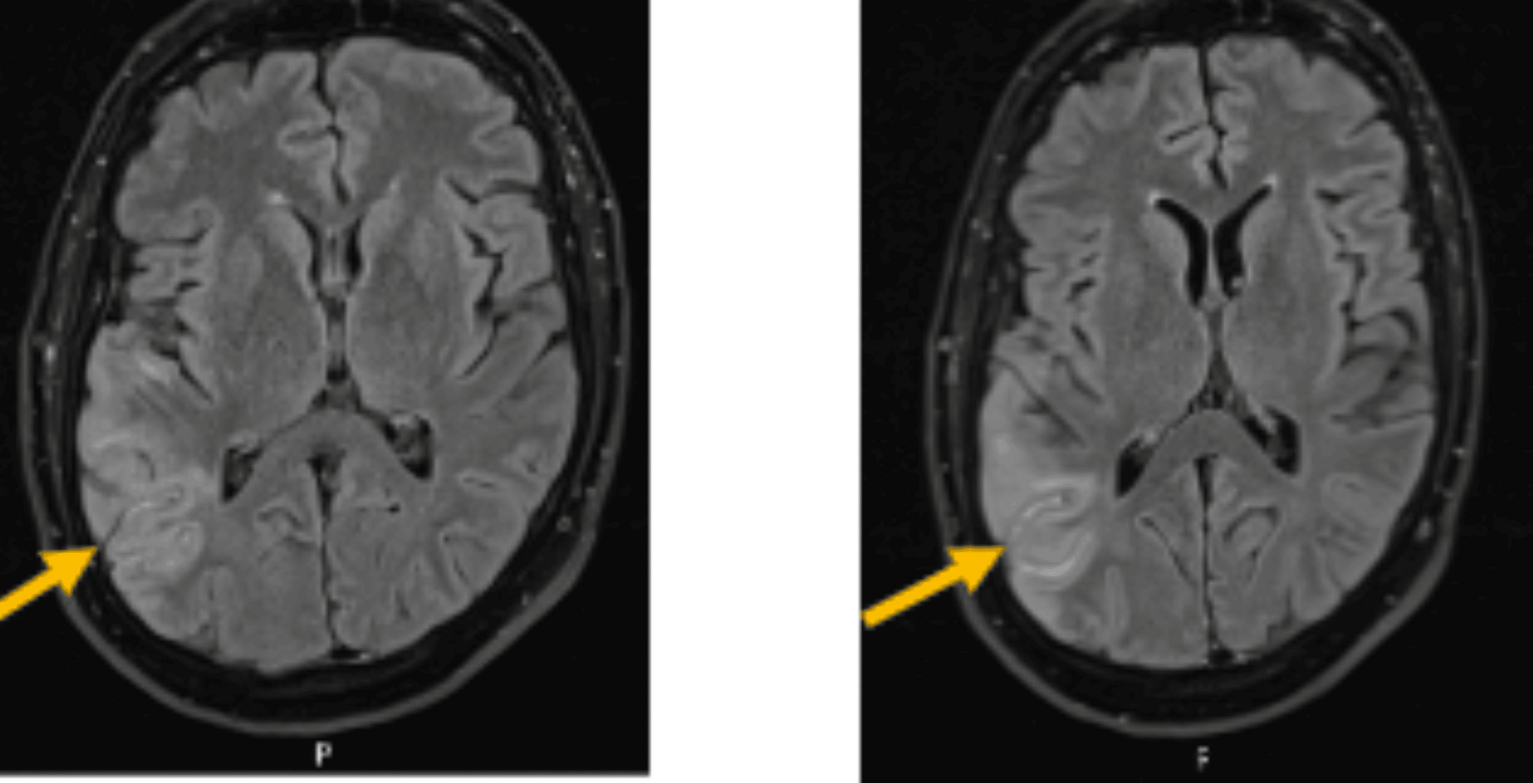
MRI head sub-acute right MCA territory infarct. Area of increased FLAIR signal involving the cortex and subcortical white matter of the posterior right parietal and right temporal lobes. MCA: middle cerebral artery; FLAIR: fluid-attenuated inversion recovery

Ten days later, he re-presented with acute expressive dysphasia. On examination, he was haemodynamically stable but had a new early diastolic murmur best heard at the left sternal edge. Neurological examination demonstrated expressive aphasia without motor weakness. CT and MRI of the brain confirmed a subacute infarct in the right middle cerebral artery territory (Figure 1).

**Figure 2:**
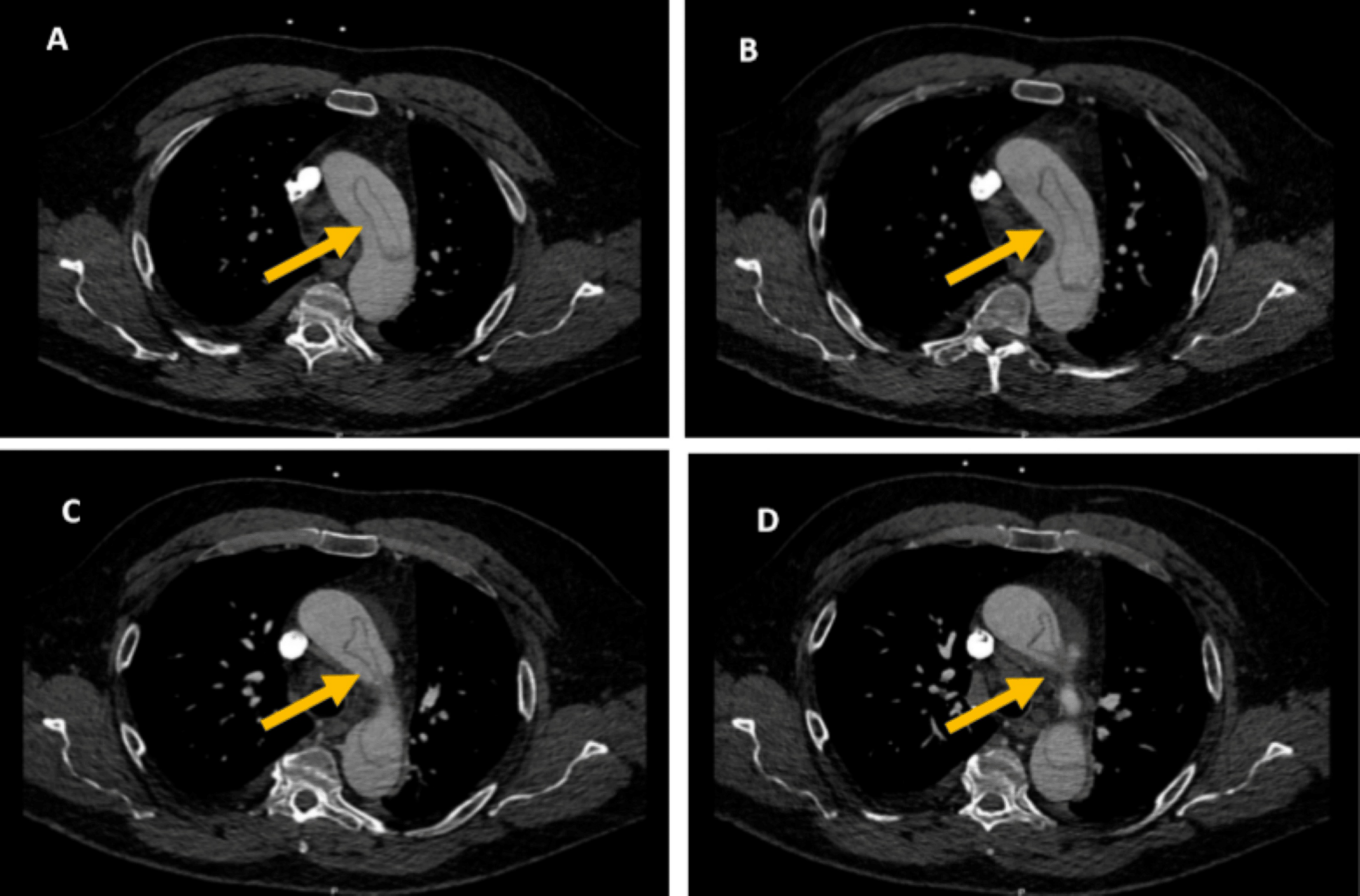
CT angiogram of the aorta showed a Stanford A aortic dissection arising from the aortic sinus and terminating in the infra-renal abdominal aorta. Extension into the brachiocephalic and left subclavian arteries is seen, with the RCA, coeliac, superior and inferior mesenteric arteries arising from the false lumen. The aortic root was dilated to 5.5 cm. RCA: right coronary artery

Given the history of preceding severe chest pain, transient altered consciousness, and the newly detected murmur, transthoracic echocardiography was performed. This demonstrated features concerning for aortic root pathology, including a dilated ascending aorta and a suspected intimal flap. CT aortic angiography subsequently confirmed a Stanford type A aortic dissection (Figure 2). The patient underwent urgent surgical repair.

Postoperatively, he was admitted to the intensive care unit for mechanical ventilation and vasopressor support. His recovery was complicated by *Escherichia (E.) coli *sepsis requiring intravenous antibiotics. Despite treatment, he deteriorated and passed away; the cause of death was attributed to *E. coli *sepsis following surgical repair of a Stanford type A aortic dissection.

## Discussion

A Stanford type A aortic dissection is a life-threatening condition that remains challenging to diagnose due to its variable clinical presentation and frequent absence of classical findings on initial evaluation [10]. While sudden, severe chest pain is the most common symptom, up to one-third of patients present with atypical features, such as syncope or acute neurological deficits, contributing to delayed or missed diagnoses [11]. This case highlights key clinical red flags, including severe chest pain, transient altered consciousness, and subsequent ischaemic stroke, which should prompt early consideration of aortic dissection despite initially normal investigations [5].

Early cardiothoracic surgical involvement is critical, as untreated Stanford type A dissection carries a high early mortality rate, increasing by approximately 1-2% per hour in the first 48 hours [12]. CT aortic angiography remains the diagnostic gold standard [13]. Bedside transthoracic echocardiography may provide rapid supportive evidence, while D-dimer has high sensitivity in early presentations and may aid risk stratification, although it lacks specificity and should not delay definitive imaging and management [14].

## Conclusions

This case illustrates the diagnostic difficulty of Stanford type A aortic dissection presenting with atypical symptoms and delayed neurological complications. It reinforces the need for a high index of suspicion in patients presenting with chest pain accompanied by transient altered consciousness or subsequent ischaemic stroke, even when initial investigations are unremarkable. Incorporating bedside echocardiography and selective use of D-dimer into early assessment may aid risk stratification, but definitive diagnosis and management planning depend on prompt CT aortic angiography and early cardiothoracic surgical involvement.
